# A Case Report on May-Thurner Syndrome: Beyond the Usual Suspects

**DOI:** 10.7759/cureus.48837

**Published:** 2023-11-15

**Authors:** Jorge Nadal Bosch, Mario Moya, Samuel Serna, Richard Sanchez, Javier Malcolm

**Affiliations:** 1 Diagnostic Radiology, Doctors Hospital at Renaissance, Edinburg, USA; 2 Radiology, Doctors Hospital at Renaissance, Edinburg, USA; 3 Cardiology, Doctors Hospital at Renaissance, Edinburg, USA; 4 Medical Information, Doctors Hospital at Renaissance, Edinburg, USA

**Keywords:** may-thurner syndrome, interventional radiology stent placement, deep vein thrombosis (dvt), balloon dilation, mechanical thrombectomy (mt)

## Abstract

May-Thurner syndrome (MTS), also known as iliocaval venous compression syndrome, is a vascular condition characterized by extrinsic venous compression within the iliocaval territory. While traditionally considered a condition predominantly affecting women, this case report presents an atypical presentation in a middle-aged male patient. The patient initially presented with left lower extremity pain and swelling, which was attributed to deep venous thrombosis (DVT) in the left calf and femoral vein. Despite anticoagulation therapy, his symptoms persisted, leading to further diagnostic evaluation and the identification of MTS. This report highlights the clinical presentation, diagnostic challenges, and successful management of MTS in a male patient. Endovascular interventions, including balloon dilation and stent placement, were employed to address refractory stenosis and thrombus burden. The case emphasizes the importance of considering MTS as a potential diagnosis in patients with unexplained lower limb symptoms, irrespective of traditional risk factors or gender. Early identification and appropriate interventions can lead to symptom relief, obstruction resolution, and improved long-term outcomes for patients with MTS. This case underscores the need for heightened clinician awareness regarding MTS and its potential impact on patient care.

## Introduction

May-Thurner syndrome (MTS), also recognized as iliocaval venous compression syndrome, Cocket's syndrome, and venous spur, is a vascular condition characterized by extrinsic venous compression within the iliocaval territory, primarily due to the arterial system exerting pressure against bony structures in this region [1]. While MTS can remain asymptomatic, it has the potential to progress and manifest as symptoms associated with venous occlusion and venous hypertension, occasionally culminating in deep venous thrombosis (DVT) [2]. It is worth noting that the true prevalence of MTS remains uncertain due to underdiagnosis, thereby suggesting that its incidence may be higher than currently reported in the medical literature [3-4].

MTS represents a relatively infrequent etiology of DVT, contributing to approximately 2-5% of DVT cases, and it exhibits a noticeable predilection for occurrence among women compared to men [5-6]. Interestingly, male patients with MTS often present with symptoms characterized by heightened pain and swelling in the affected lower extremity, while female patients more frequently present with pulmonary embolism, and this presentation typically occurs at a younger age. In this context, we present a clinical case involving MTS in a middle-aged male patient who presented with left lower extremity pain and swelling, highlighting the need for vigilant clinical assessment and diagnostic evaluation in suspected cases of MTS.

## Case presentation

This is a 63-year-old Hispanic gentleman with no significant past medical history who presented to the Emergency Department (ED) with a chief complaint of left lower extremity swelling and pain that had been ongoing for one week. Prior to this ED visit, he had consulted his primary care physician (PCP) and was prescribed rivaroxaban. Upon arrival at the ED, his vital signs were stable, and he denied any associated symptoms, such as chest pain and shortness of breath. However, the patient was noted to be in moderate distress, primarily due to the pain.

**Figure 1:**
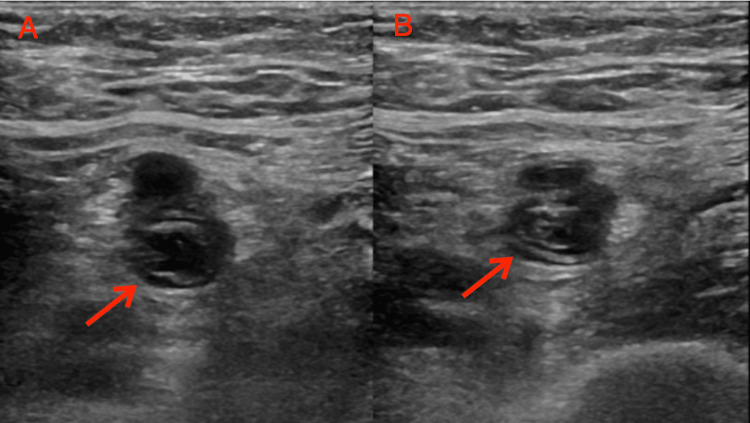
Venous Doppler ultrasound of the left lower extremity showing a deep vein thrombosis of the left femoral vein. Image A illustrates a typical depiction of the femoral artery and femoral vein alongside deep vein thrombosis, while Image B presents the same arterial and venous structures afflicted by deep vein thrombosis, with a notable absence of venous compression despite attempted compression techniques.

Physical examination revealed an erythematous left lower extremity with tenderness upon palpation and edema. Lower extremity Doppler ultrasonography was performed, showing an occlusive DVT in the left calf, extending to include the left femoral vein, as well as the involvement of the superficial small saphenous and great saphenous veins (Figure 1). Furthermore, chronic occlusive DVT was identified in the right popliteal veins.

The medical team sought the expertise of the Interventional Radiology (IR) team and collectively determined to proceed with the venogram procedure, which confirmed the presence of popliteal vein thrombosis and outlined the need for further intervention.

IR and the medical team elected to proceed with a local infusion of a thrombolytic agent. The procedure began with the access of the vein using a micro-puncture set. A guidewire was then advanced into the femoral vein, followed by the administration of contrast, which clearly demonstrated complete thrombosis from the popliteal vein extending into the femoral vein and common femoral vein. Subsequently, a 6 French 10 cm Terumo sheath was successfully placed after advancing the guidewire into the inferior vena cava (IVC). A 5 French Kumpe catheter was introduced into the left common femoral vein through the sheath. The venogram obtained during this phase confirmed the diagnosis of MTS, with patent distal IVC and the catheter tip in the IVC. To facilitate treatment, a 100 cm length 50 cm infusion length McNamara catheter was placed, with its proximal side hole positioned in the area of Hunter's canal. An overnight infusion of tissue plasminogen activator (tPA) at 1 mg per hour was initiated via the infusion catheter. Following the procedure, the patient was transferred to the intensive care unit (ICU) for post-procedural care.

**Figure 2:**
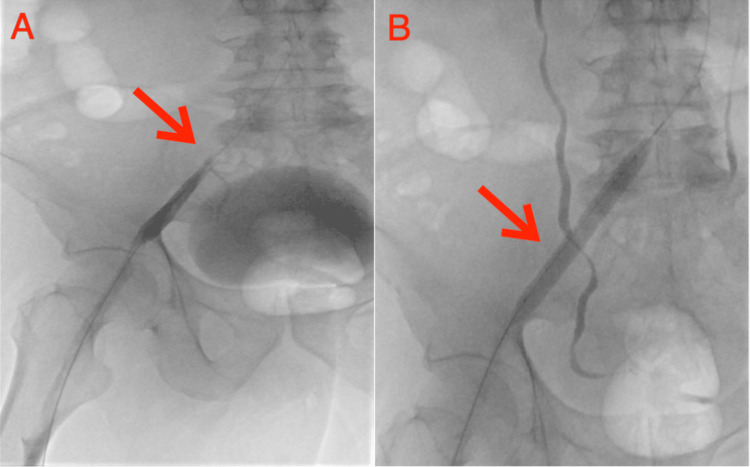
Venography of the left iliac vein. Image A reveals a demonstrative compression of the left iliac vein by the right iliac artery, unequivocally confirming the presence of May-Thurner syndrome. In stark contrast, image B captures the inflation of the balloon catheter within the vascular lumen dilating the left iliac vein.

On hospital day one, the patient's left lower extremity symptoms persisted, prompting a second left lower extremity venogram and cavogram. This revealed persistent thrombus within the left superficial, common femoral, external iliac, and common iliac veins, although the popliteal and distal superficial femoral artery remained patent. Notably, there was interval improvement in clot burden with no significant stenosis at the level of the proximal common iliac vein. However, a severe stenosis of 75% was identified at the left common femoral and iliac veins. The IR team, in collaboration with the medical team, reached a consensus to proceed with mechanical thrombectomy, which was performed at the left superficial femoral, common femoral, and iliac veins using a 10 mm balloon. Despite initial balloon dilation of the left common femoral vein stenosis, refractory stenosis persisted, with significant residual stenosis of more than 50% (Figure 2).

Consequently, a 10 mm drug-eluting balloon was employed, resulting in interval improvement in flow and caliber, with a residual stenosis of 30%.

**Figure 3:**
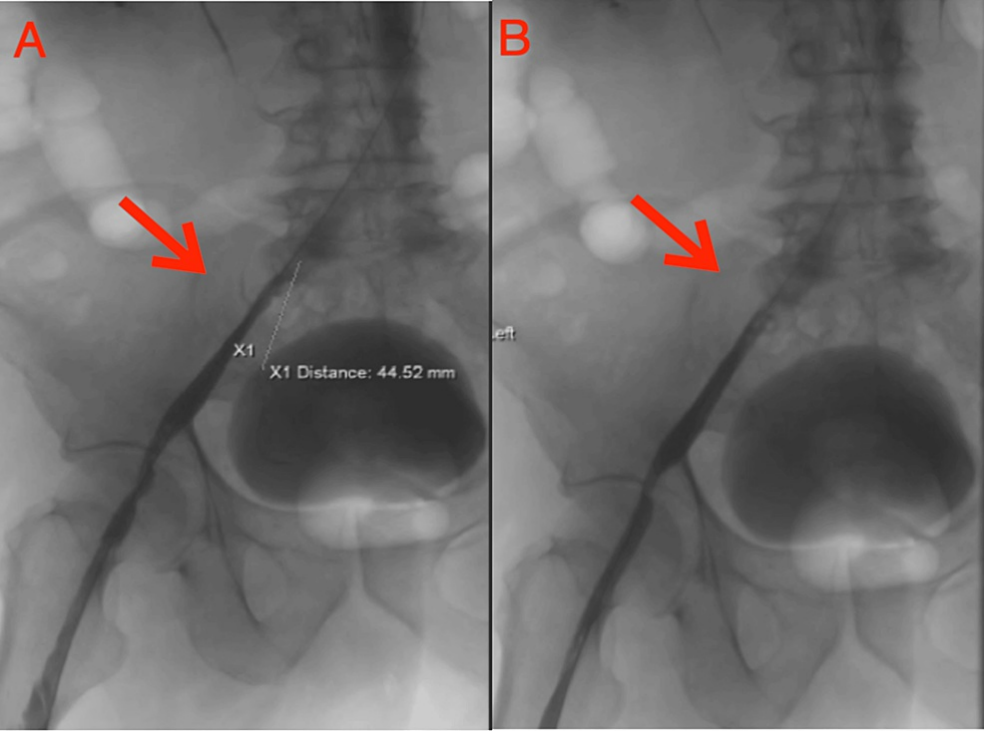
Venogram showing a 12 x 60 mm Life Star self-expanding stent being deployed within the left iliac vein. Image A depicts a venogram immediately following the deployment of a stent in the left iliac vein with the cranial end of the stent located approximately 44.52 mm from the IVC bifurcation, which was previously subjected to compression by the right iliac artery. In contrast, Image B illustrates the post-stent deployment scenario, demonstrating the complete resolution of stenosis within the left iliac vein.

Further, a 12 mm balloon was used to dilate the left iliac vein stenosis; however, stenosis refractory to previous dilations persisted, with a residual stenosis of more than 50%. To address this, a 12 x 60 mm Life Star self-expanding stent was deployed within the left iliac vein, centered on the area of critical stenosis (Figure 3).

Subsequent balloon dilation with a 12 mm balloon demonstrated interval improvement in flow and caliber, with no significant residual thrombus. Following these interventions, the patient was transferred to the medical unit, where he received continued care on a heparin drip.

On the third day of admission, the patient reported symptomatic improvement, and he was discharged home on chronic anticoagulation with rivaroxaban.

## Discussion

MTS is a progressive vascular condition characterized by the compression of the left iliac vein by the right common iliac artery, typically occurring at the fifth lumbar vertebra [7]. This compression leads to an increased risk of various long-term complications, emphasizing the clinical significance of early detection and intervention. One of the primary concerns associated with MTS is the development of lower extremity swelling, which can significantly impact a patient's quality of life. The compromised blood flow resulting from the compression may also predispose individuals to the formation of DVT, a serious condition where blood clots form in the deep veins, posing a risk of migration to the lungs and causing pulmonary embolism a potentially life-threatening complication.

In addition to DVT and pulmonary embolism, MTS is implicated in the pathogenesis of chronic venous stasis ulcers. These ulcers are often challenging to manage, requiring a multidisciplinary approach for effective treatment. The chronic venous insufficiency resulting from MTS can lead to skin changes, subcutaneous fibrosis, and impaired tissue healing, contributing to the development of ulcers. Furthermore, MTS has been associated with phlegmasia cerulea dolens, a severe form of deep venous thrombosis characterized by extensive swelling, cyanosis, and impaired arterial blood flow [8]. Prompt diagnosis and intervention are crucial to prevent the progression of MTS and mitigate the associated complications.

Clinical suspicion of MTS typically hinges on observed clinical features and initial diagnostic evaluations, often initiated with a duplex ultrasound [9]. To establish a definitive diagnosis of MTS, vascular imaging must reveal the presence of a stenotic or acoustic venous lesion. Diagnostic criteria also encompass evaluating the severity of the condition and the presence of DVT.

The selection of an appropriate treatment strategy for MTS necessitates a meticulous assessment of the patient's individual symptoms, the overall severity of the disease, and the concurrent presence of DVT [10]. Emphasizing the critical importance of tailoring interventions to align with the unique clinical presentation of each patient is imperative in the context of medical management. In the case described herein, despite the patient undergoing multiple balloon dilation attempts and mechanical thrombectomy, refractory residual stenosis persisted. This refractory nature of the stenosis prompted a comprehensive evaluation of available treatment options, leading to the decision to proceed with stent placement.

Medical literature has increasingly recognized endovascular interventions, such as stent placement, as valuable components of the therapeutic arsenal against MTS. A growing body of evidence, spanning studies highlighting the efficacy of stent placement in alleviating MTS-related symptoms, underscores its role in addressing residual stenosis, optimizing flow dynamics, and achieving the absence of residual thrombus. The positive outcomes observed in this case underscore the importance of considering endovascular interventions, particularly stent placement, in cases of MTS where conventional treatments may prove insufficient [10-12].

## Conclusions

This case report highlights the atypical yet clinically significant presentation of MTS in a 60-year-old male, challenging the conventional demographic expectations associated with this vascular condition. Despite its common occurrence in females aged 20-50, the successful diagnosis and subsequent treatment underscore the importance of considering MTS in diverse patient populations. This case emphasizes the necessity for clinicians to maintain a broad differential diagnosis, irrespective of age or gender norms, to ensure timely and effective intervention. The favorable outcome achieved through stent placement reinforces the importance of individualized and comprehensive management strategies in addressing MTS, particularly when presented outside the typical demographic parameters. Continued research and clinical trials are essential to further refine the understanding of optimal treatment modalities for MTS, taking into account factors such as long-term outcomes, recurrence rates, and comparative effectiveness. This evolving landscape of medical literature contributes to the ongoing improvement of evidence-based guidelines, providing clinicians with the knowledge needed to make informed decisions tailored to the specific nuances of each MTS case. Continued documentation of such cases contributes to a deeper understanding of the varied clinical manifestations of this syndrome and further enriches the medical literature for improved patient care.
